# Intravenous Cardiac Stem Cell-Derived Exosomes Ameliorate Cardiac Dysfunction in Doxorubicin Induced Dilated Cardiomyopathy

**DOI:** 10.1155/2015/960926

**Published:** 2015-08-17

**Authors:** Adam C. Vandergriff, James Bizetto Meira de Andrade, Junnan Tang, M. Taylor Hensley, Jorge A. Piedrahita, Thomas G. Caranasos, Ke Cheng

**Affiliations:** ^1^Molecular Biomedical Sciences, College of Veterinary Medicine, North Carolina State University, Raleigh, NC 27695, USA; ^2^Joint Department of Biomedical Engineering, UNC-Chapel Hill and NC State University, Chapel Hill and Raleigh, NC, USA; ^3^Department of Cardiology, First Affiliated Hospital, College of Medicine, Zhengzhou University, Zhengzhou, Henan 450052, China; ^4^Division of Cardiothoracic Surgery, University of North Carolina at Chapel Hill, Chapel Hill, NC 27599, USA; ^5^The Cyrus Tang Hematology Center, Soochow University, Suzhou 215123, China

## Abstract

Despite the efficacy of cardiac stem cells (CSCs) for treatment of cardiomyopathies, there are many limitations to stem cell therapies. CSC-derived exosomes (CSC-XOs) have been shown to be responsible for a large portion of the regenerative effects of CSCs. Using a mouse model of doxorubicin induced dilated cardiomyopathy, we study the effects of systemic delivery of human CSC-XOs in mice. Mice receiving CSC-XOs showed improved heart function via echocardiography, as well as decreased apoptosis and fibrosis. In spite of using immunocompetent mice and human CSC-XOs, mice showed no adverse immune reaction. The use of CSC-XOs holds promise for overcoming the limitations of stem cells and improving cardiac therapies.

## 1. Introduction

Cardiac stem cells (CSCs), such as cardiosphere-derived cells and c-kit (CD117)+ cells, have been shown to be an effective treatment for ischemic heart disease [1] including the CADUCEUS and SCIPIO clinical trials [2, 3]. Despite the effectiveness of CSC injection, several limitations remain that may hinder the transition from the lab to the clinic [4, 5]. Autologous injection of CSCs requires that the patient wait 4–6 weeks for cell manufacturing to be completed. Though allogeneic cell therapy has shown to be effective [6], there still remains a risk of cell rejection and inflammation. In addition to the compatibility issues, cell therapies usually require the use of intramyocardial (IM) or intracoronary (IC) injection to avoid the “first pass effect” in which intravenously (IV) delivered cells become trapped in the lungs [7] or other organs such as the spleen [8]. Even using IM or IC injection [9], the cell retention rates in the heart can be as low as 11% and 2.6%, respectively [10]. Of the small population of cells that reach the heart, these cells fail to engraft and will eventually die [6].

Recent research has shown that a large portion of the beneficial effects of stem cell therapies come from paracrine effects [11]; that is, injected cells secrete microvesicles, proteins, and microRNAs to promote endogenous regeneration. One such cellular secretion is lipid bilayer particles known as exosomes [12, 13]. Exosomes contain proteins and microRNAs (miRNAs) [14] that can alter cellular activity in the target cells. CSC-derived exosomes (CSC-XOs) have been shown to regenerate cardiac function in murine models of myocardial infarction [15, 16], yet the CSC-XOs were delivered intramyocardially via an open-chest surgery.

Previous research has shown that CSCs are also effective in the treatment of dilated cardiomyopathy (DCM) [17, 18]. The objective of this study is to test the efficacy of intravenous CSC-XOs for the treatment of dilated cardiomyopathy. Due to the small size of the exosome, we also anticipate that exosomes are not subject to excessive entrapment within the lung or other organs, and IV delivery will result in safe and therapeutically effective amounts of exosomes being delivered to the heart. Finally, through the use of human CSC-XOs, we will show whether xenogeneic injection of exosomes yields any immune response in mice.

## 2. Methods

### 2.1. Cell Culture and Exosome Isolation

Cardiac stem cells (CSCs) were generated and cultured using the cardiosphere method as previously described [2, 15]. In brief, passage 4 hCSCs were cultured in IMDM containing 20% fetal bovine serum (FBS) until 90% confluency; then medium was switched to serum-free Iscove's Modified Dulbecco's Medium (IMDM). Medium was conditioned by the CSCs for 15 days, then removed, and stored at −80°C awaiting further processing.

For exosome isolation, CSC-conditioned medium was thawed slowly on ice overnight; then exosomes were isolated using ultrafiltration [19]. Briefly, 10 mL exosome conditioned medium was filtered through 0.22 *μ*m vacuum sterilization filters (Millipore, Billerica, MA) to remove larger vesicles and cellular debris. The filtrate was then added to Amicon Ultra-15 100 kDa filters (Millipore, Billerica MA) and spun at 4000 ×g for 10 min. Labeling was performed using 10 *μ*M DiI (Life Technologies, Carlsbad, CA) for 30 min. Filtrate was discarded, and volume of retentate was adjusted to 15 mL with PBS. Spinning and washing with PBS was repeated two more times. On the last cycle, remaining volume containing the exosomes (approximately 1 mL) was transferred to micro centrifuge tube and stored at −20°C.

### 2.2. Flow Cytometry Analysis

Flow cytometry was performed on hCSCs using a LSR-II (Beckman-Dickenson, Franklin Lakes, NJ). Data was analyzed using FlowJo (Treestar, Ashland, OR). Cells were incubated with antibodies for CD105 (fab10971p, R&D Systems, Minneapolis, MN), CD90 (BD555595, BD, Franklin Lakes, NJ), c-kit (BD550412, BD), and CD45 (BD555482, BD) for 60 min. Isotype-identical antibodies (BD559320, BD555748, and BD555751, BD) were used as negative controls.

### 2.3. Exosome Characterization

Exosome size and concentration were determined using nanoparticle tracking analysis (NTA; NanoSight, Malvern, UK). 10 *μ*L of exosome sample was diluted into 990 *μ*L PBS to achieve proper concentration for NTA. Samples were imaged five times for 90 sec and analyzed. Exosome immunofluorescence images were taken using methods previously described [20].

### 2.4. *In Vitro* Study

Neonatal rat cardiomyocytes (NRCMs) were isolated, cryopreserved, and then cultured as previously described [21] in 4-well culture slides. After culturing for 3 days, culture medium was supplemented with 0.7 × 10^9^ DiI-labelled hCSC-exosomes, cultured for an additional 24 hr, and then fixed with 4% paraformaldehyde and stored at −20°C for immunofluorescent staining for *α*-sarcomeric actinin.

### 2.5. Animal Studies

All animal procedures were approved by the Institutional Animal Care and Usage Committee. 6–8-week-old male CD-1 mice (Charles River Labs, Wilmington, MA) were used. 5 mg/kg body weight of doxorubicin was delivered by intraperitoneal injection to induce dilated cardiomyopathy. The mice were housed in the animal facility for 7 days to allow for complete induction of DCM. Mice were then injected with either 200 *μ*L PBS or 200 *μ*L PBS containing 30 × 10^9^ CSC exosomes via the tail vein.

### 2.6. Echocardiography

Following induction of anesthesia with isoflurane, hearts were imaged using a Philips Cx-70 Ultrasound System with a L15-7io high frequency probe. Each measurement was performed several times and then averaged. Two-dimensional guided M-mode images at chordae tendineae level were evaluated. M-mode measurements of left ventricle end-diastolic and end-systolic dimensions (LVEDD and LVESD, resp.) were performed by using the leading-edge method of the American Society of Echocardiograph [22]. For estimation of each parameter, the average of three measurements from three different cycles in an image was obtained. Left ventricular end-diastolic and systolic volumes (LVEDV and LVESV, resp.) were calculated by the biplane method of disks (modified Simpson's rule). Ejection fraction (EF) was determined by using (LVEDV − LVESV/LVEDV) × 100 and fractional shortening (FS) was calculated from the M-mode echocardiography images as (LVEDD − LVESD/LVEDD) × 100.

### 2.7. Heart Histology

Following sacrifice, hearts were removed, laterally bisected, and equilibrated with increasing sucrose solutions up to 30% overnight. Hearts were then embedded in Optimal Cutting Temperature (OCT) compound (Tissue-Tek, Torrance, CA), snap-frozen in liquid nitrogen, and then cryosectioned with a thickness of 5 *μ*m.

Haematoxylin and eosin staining (H&E) was performed using traditional methods and chemicals (Sigma-Aldrich, St. Louis, MO). Masson's Trichrome staining was performed using HT15 Trichrome Staining (Masson) Kit (Sigma-Aldrich, St. Louis, MO). Heart sections were then fixed in 4% paraformaldehyde solution, permeabilized with 0.01% saponin (Sigma-Aldrich, St. Louis, MO), and blocked using Protein Block solution (Dako, Glostrup, Denmark). Primary antibodies were incubated overnight at 4°C, and subsequently secondary antibodies at room temperature for 1.5 hr. Sections treated with TUNEL (Roche, Basel, Switzerland) were incubated for 30 minutes following the secondary antibody incubation. Samples were then treated with DAPI (LifeTech, Carlsbad, CA) and mounted in Prolong Gold Mounting Media (LifeTech, Carlsbad, CA).

### 2.8. Statistical Analysis

All statistical analysis was performed using Graphpad Prism (Graphpad Software, La Jolla, CA). Two-sided *t*-tests were performed for all analyses. *p* < 0.05 was considered significant and indicated with a single asterisk, and error bars represent standard deviation. Comparisons between two groups were performed using a student's *t*-test, and comparisons between more than two groups were performed using two-way ANOVA followed by* post hoc* comparisons with Bonferonni correction.

## 3. Results and Discussion

### 3.1. Cell Phenotype, Exosome Characterization, and* In Vitro* Study

**Figure 1:**
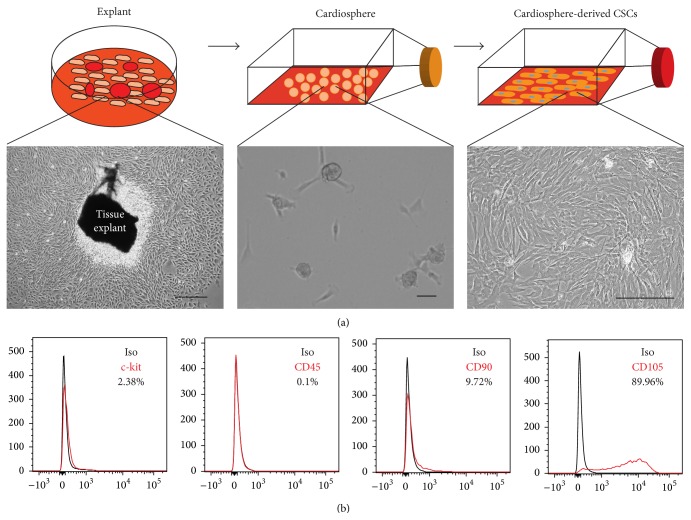
(a) Outline of the culture process going from heart biopsy, explants, cardiospheres, and finally CSCs. (b) Phase contrast images of cells at various stages of the culture process. Scale = 100 *μ*m.

**Figure 2:**
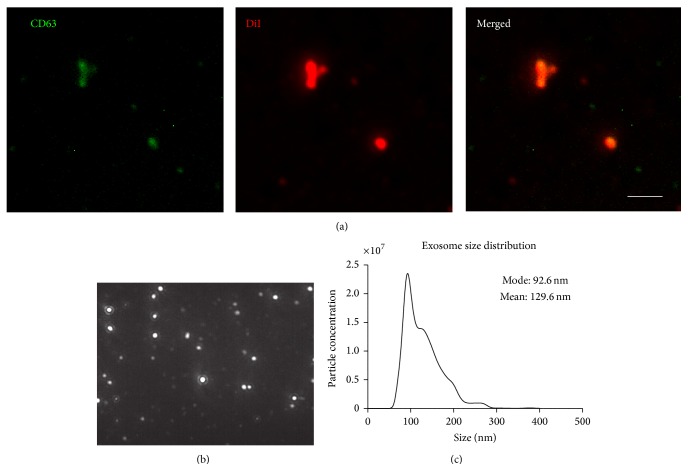
Characterization of exosomes using (a) immunofluorescence indicates the CSC-XOs are positive for CD63, an exosomal marker. Scale = 5 *μ*m. (b) NanoSight particle tracking image; (c) size distribution as reported by NTA shows that the exosomes are within the expected size range verifying there are no other extracellular vesicles present.

**Figure 3:**
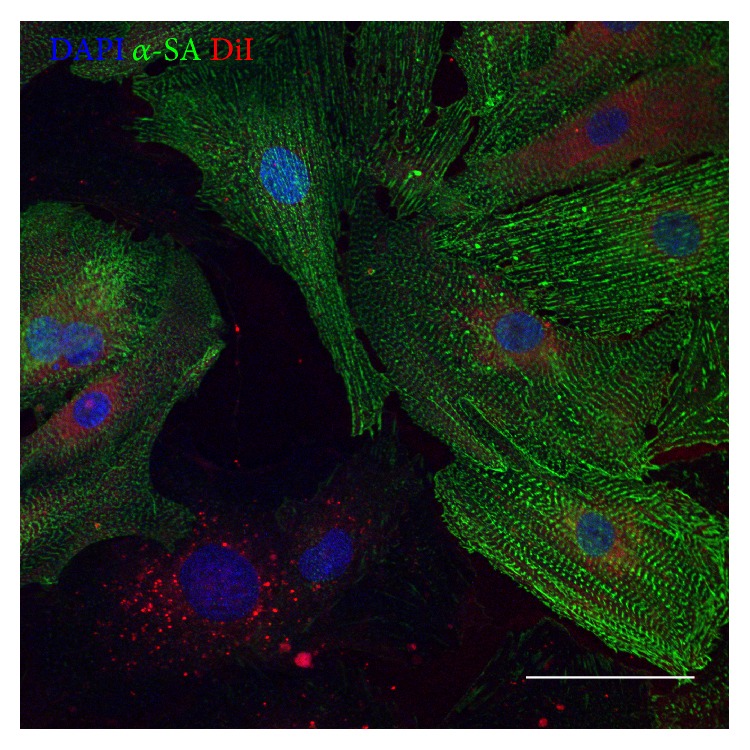
NRCMs cultured* in vitro* uptake DiI-labeled hCSC-XOs (red). The CSC-XOs can be seen within the cytoplasm of the cardiomyocytes (green). Scale = 50 *μ*m.

Microscopic analysis (Figure 1(a)) and flow cytometry (Figure 1(b)) confirmed the morphology and phenotype of cardiosphere-derived CSCs [2, 23, 24]. In order to confirm that the isolated particles were exosomes and not other cellular secretions such as microvesicles or apoptotic bodies, we examined both the size and expression of constitutive exosomal markers. The isolated particles expressed exosomal marker CD63 (Figure 2(a)). The size observed under the microscope appears larger than expected; this can be attributed to exosome clumping or due to the size of exosomes being less than the lateral resolution of the microscope. NTA confirmed the size of exosomes (less than 200 nm) (Figures 2(b) and 2(c)), confirming that they are indeed exosomes. Due to the ultrafiltration method used for exosome purification, there may be contamination with proteins over 100 kDa in size, though even other methods of isolation such as ultracentrifugation or polymer precipitation result in protein contamination as well [25]. When DiI-labeled exosomes were added to media for NRCMs, they were readily absorbed by the cells (Figure 3). The uptake of CSC-XO by cardiomyocytes warrants the potential to use them for therapeutic regeneration in DCM.

### 3.2. IV-Delivered CSC-XOs Promote Recovery of Heart Function

**Figure 4:**
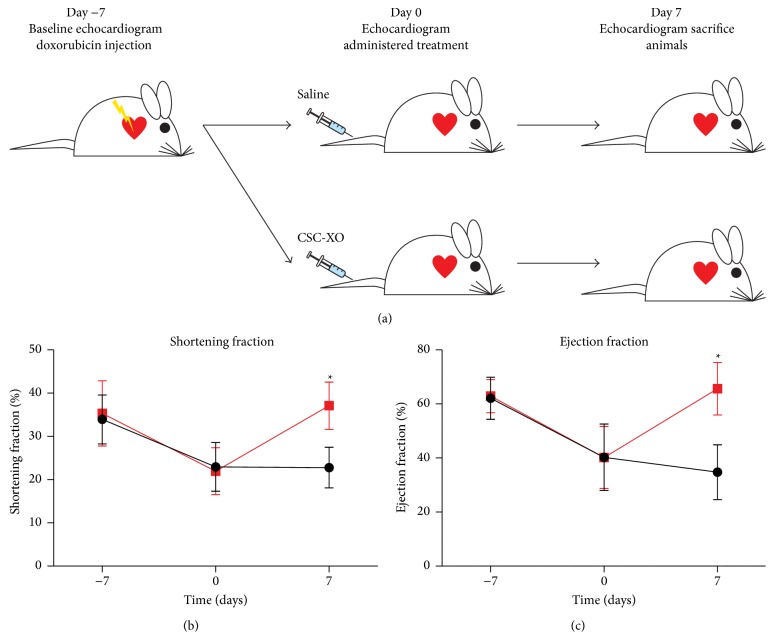
(a) Outline of the* in vivo* portion of the study. Heart function analysis showing both the (b) shortening fraction and (c) ejection fraction. Both measurements of heart function showed improvement in the CSC-XO treated groups 7 days following CSC-XO injection.

An acute model of doxorubicin induced DCM was used for the animal study (Figure 4(a)). Seven days after injecting mice with doxorubicin, heart function decreased significantly from the baseline measurements. The animals receiving CSC-XO treatment showed a recovery of heart function (Figures 4(b) and 4(c)) whereas the animals receiving saline injections continued with cardiac functional deterioration. In a previous study by Aminzadeh et al. [17], a similar study was carried out but with several key differences. IM transplantation of CDCs was tested in G*α*q mice, which spontaneously develop DCM. Their results showed a steady albeit decreased heart function as opposed to the current study which showed a recovery of heart function to the baseline levels. In addition, IM injection is quite invasive because open-chest procedures are normally needed and thus would be difficult for clinical translation.

### 3.3. CSC-XOs Reduce Apoptosis and Fibrosis in the Heart

**Figure 5:**
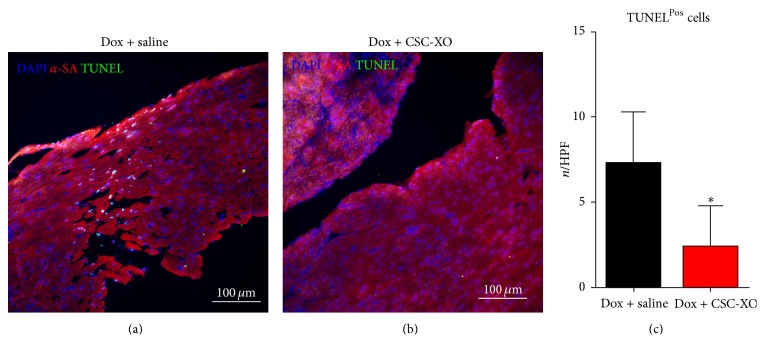
Comparison of TUNEL staining of specimens receiving saline (a) or CSC-XO (b). Counting total amount of TUNEL^Pos^ cells in each field of view indicates a significant drop in apoptotic cells (c).

**Figure 6:**
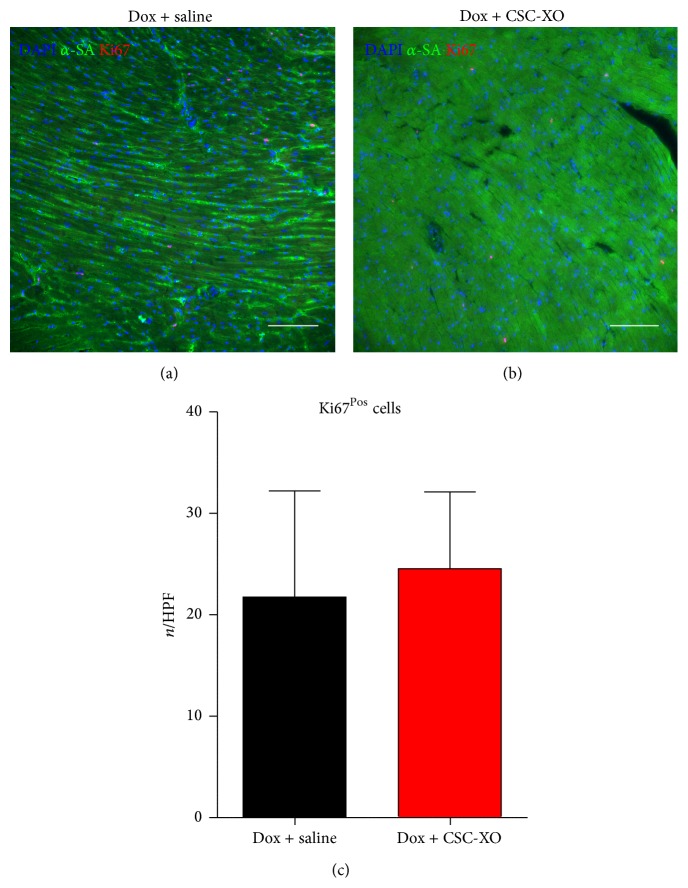
Cellular proliferation as indicated by Ki67^Pos^ cells for specimens receiving saline (a) or CSC-XO (b) shows no significant changes in number of positive cells per field of view (c). Scale = 100 *μ*m.

**Figure 7:**
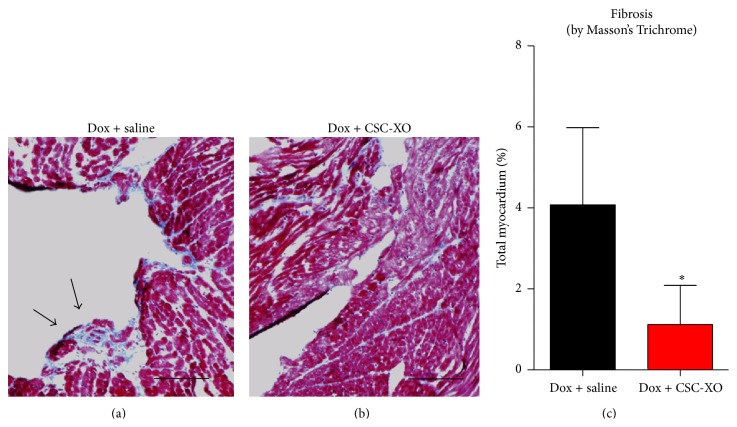
Masson's Trichrome staining of heart sections from specimen receiving saline (a) and animals receiving CSC-XO (b) shows a decrease in the area of fibrotic lesions (c). Scale = 100 *μ*m.

In order to verify that disease-modifying changes were occurring at the cellular level, we performed histological analyses of the heart sections. hCSC-XOs have been shown to carry miRNAs (22, 24, 146, and 210) that decrease fibrosis by inhibiting TGF-*β* signaling pathways [15] and reduce apoptosis [16]. Detection of apoptosis was carried out with TUNEL, which showed a significant reduction in the CSC-XO treated hearts (Figure 5). Subsequently, we examined cellular proliferation through Ki67. In both groups there were numerous Ki67^Pos^ cells (~23/HPF), yet there was no significant difference between the groups (Figure 6), indicating CSC-XO did not promote cardiomyocyte cycling. DCM does induce large amounts of fibrotic growth in the heart, which reduced contractility and function. Masson's Trichrome staining revealed that CSC-XO treatment decreases cardiac fibrosis (Figure 7). Previous studies have shown that CSC-XO is enriched with a variety of microRNAs which could have inhibited the apoptosis and fibrosis pathways in the post-MI heart.

### 3.4. Xenogeneic CSC-XOs Induce Minimal Immune Response

**Figure 8:**
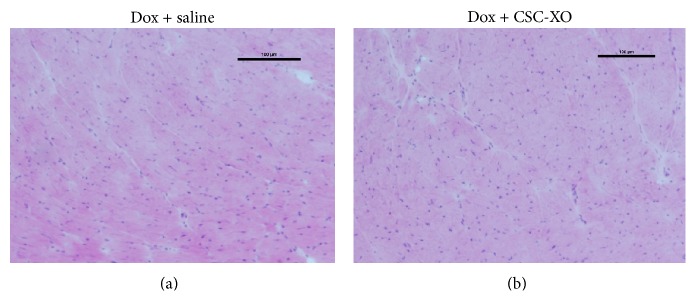
H&E staining of heart sections. Lack of cellular infiltrations indicates no negative immune response by mice.

Using H&E staining, there was little infiltration shown in animals receiving human exosomes compared to the control group (Figure 8), suggesting CSC-XO did not elicit measurable immune response. A lack of immune response from xenogeneic translation indicates that using allogeneic transplantation in humans is highly probable. Despite the lack of immune response in the heart, other organs were not examined for any immune response. Future studies are needed to examine other organs and systemic immune response to allogeneic exosome therapies. Nevertheless, if allogeneic transplantation is safe, it allows for exosomes to be collected from controlled cell lines that are known to stably produce therapeutic exosomes, as well as allowing for the exosomes to be pulled “out of the shelf” for acute cardiac events.

## 4. Conclusions

The research carried out confirms that CSC-XOs are capable of regenerating cardiac function in nonischemic diseases such as DCM. In addition, we have shown that IV delivery of exosomes is effective, which reduces the invasiveness of exosome delivery to a minimum. Finally, we verified the lack of immune response of xenogeneic injection of CSC-XOs. This result of this study verifies that CSC-XOs are effective in the treatment of DCM. CSC-XOs offer great promise for clinical treatments as they mitigate many of the limitations of stem cell therapy such as delivery and storage.
